# Editorial: Quality of sexual and reproductive health care: strengths, gaps, and challenges for midwifery care

**DOI:** 10.3389/fgwh.2025.1546264

**Published:** 2025-02-06

**Authors:** Lorena Binfa, Loreto Pantoja, Ramon Escuriet

**Affiliations:** ^1^ Faculty of Medicine, Department of Women’s and Newborn Health, University of Chile, Santiago, Chile; ^2^ GHenderS Research Group, Blanquerna School of Health Sciences, University Ramon Llull, Barcelona, Spain

**Keywords:** sexual and reproductive health, midwifery, quality of care, training and education, maternal and newborn care

**Editorial on the Research Topic**
Quality of sexual and reproductive health care: strengths, gaps, and challenges for midwifery care

## 1 Introduction

Sexual and Reproductive Health (SRH) extends beyond traditional biological and medical approaches to encompass a broader range of human needs to achieve comprehensive sexual and reproductive well-being. Such well-being is attained when individuals demand and exercise their rights (1). The concept of SRH addresses the entire life cycle of all individuals, with emphasis on critical periods such as birth, adolescence, and reproductive age, along with the influence of sex and gender. Its impact is particularly profound in low- and middle-income countries (LMICs), where women often face heightened discrimination and challenges in accessing SRH care. Vulnerable groups include adolescents, single women, immigrants, refugees, ethnic or religious minorities, those living in poverty, those in camps or humanitarian crises, individuals with disabilities or HIV, and those affected by inequalities. Delivering high-quality sexual and reproductive care (SRC) requires humanized, integrated, efficient, and effective services with a strong focus on quality from both a technical and a user experience perspective (2–4).

Despite commitments made by countries at international conferences, such as the International Conference on Population Development (ICPD) in Cairo (1994), the Fourth World Conference on Women in Beijing (1995), and ICPD25 in Nairobi (2019), inequities in SRC access and quality persist. These efforts align with the commitments outlined in the 2030 Agenda for Sustainable Development. In 2021, the Generation Equality Forum further emphasized the aim of achieving gender equality by 2030 (Goal 5). Recent World State of Population Reports (WSoPR) have underscored these gaps, calling for action to address inequalities in sexual and reproductive health and rights across countries (5–9). Additionally, there is a growing need for research aimed at improving the quality of SRC, implementing models of care that enhance well-being and safety, optimizing physiological processes during childbirth, and validating outcome measures. This editorial proposes to address these needs as a central focus.

In total, 15 studies were selected for this Research topic, encompassing research conducted in North and South America, Africa, and Asia. These studies primarily focused on 4 of the 11 proposed themes.

### 1.1 Safe and respectful maternal and newborn care

Six articles addressed this theme, highlighting disparities between immigrant populations and native residents, high rates of cesarean sections (C-sections), and inadequate preparedness for the COVID-19 pandemic. The overuse of episiotomies, insufficiently trained professionals in the prevention of obstetric fistulas, non-consensual treatment, disrespect and abuse, poor antenatal education and monitoring, and women's lack of awareness regarding their own rights were recurring concerns (a, g, j, k, m, o). These studies, primarily conducted in the African Region and Latin America, align with findings from global research indicating that no country has consistently provided high-quality maternal and newborn care (2), particularly in Latin America (10). In 2024, the WHO released its position paper on transitioning to midwifery models of care, described as a “process of reorienting health systems away from the currently prevalent fragmented and risk-oriented model of care to a midwifery model of care in which women and newborns, starting from pre-pregnancy and continuing through the postnatal period, receive equitable, person-centered, respectful, integrated, and high-quality care, provided and coordinated by midwives working within collaborative interdisciplinary teams” (11). This initiative aims to encourage governments to value the contribution of midwives to improving the quality of SRC and to integrate them fully into health services in order to utilize their competencies effectively.

### 1.2 Key or targeted populations (refugees, migrants, adolescents, and sexual diversity)

Four articles explored this theme. They emphasized the complexity of health-seeking behaviors among Indigenous communities and the importance of addressing structural barriers and designing culturally appropriate programs. Similar recommendations were made for preventing miscarriages among Latina women living in the United States, with calls for further research on intimate partner violence, acculturation, and self-rated health perceptions. These measures aim to reduce disparities among immigrant populations and establish new regulations to safeguard their reproductive rights (b, c, f, g). These findings are consistent with those presented at the last UNFPA WSoPR (5–9). This theme underscores the critical role of midwives in resource-limited areas or settings with barriers to accessing health services. Midwives, when educated to international standards, integrated into health systems, and functioning as part of multidisciplinary teams, can significantly improve health outcomes. It is estimated that midwifery interventions could prevent 67% of maternal deaths, 64% of neonatal deaths, and 65% of stillbirths in 88 LMICs, where the majority of these deaths occur (12).

### 1.3 Promotion of SRH in the community

Nine articles addressed this topic, highlighting the importance of strengthening community-based education and tailoring counseling to meet not only women's needs but also family engagement. Additionally, the articles emphasized the significance of self-care options and mechanisms for supporting self-care users with information, counseling, and linkages to care (a, b, c, d, e, f, g, i , j, n). The inclusion of trained midwives can provide approximately 90% of primary health care for women and newborns. This includes family planning, perinatal mental health care, prevention of sexually transmitted diseases, and overall promotion of sexual and reproductive health-related well-being (13).

### 1.4 Midwifery education and training

All articles on this Research topic underscored the pivotal role of midwifery professionals in improving SRC quality. Strengthening midwifery education, providing regular in-service training, facilitating career transitions for newly qualified midwives, and improving continuing education were identified as critical. Sustained funding for midwifery education and strengthening of health systems are essential to ensure that midwives can effectively apply their skills (a, h, l, o). High-quality midwifery care necessitates evidence-based education and training. Evidence suggests that implementing the full scope of midwifery improves the majority of SRH outcomes, with 56 specific outcomes identified (14, 15). Training programs obtaining an academic degree positively impact patient outcomes and support the call for standardizing midwifery education at the bachelor's level. Advanced education enables midwives and nurses to assume broader roles, including leadership, research, and teaching in both clinical and academic settings (16–21). In 2019, Member States launched the WHO-UNFPA-UNICEF-BWI Joint Framework for Action at the World Health Assembly to strengthen quality midwifery education and achieve universal health coverage. This framework presents the latest global evidence, a consensus for action, and a seven-step action plan for quality midwifery education (14).

## 2 Conclusion

The recurrence of certain themes suggests potential for further exploration. Lessons learned by midwives and their contributions to improving SRH should be incorporated into future strategies. Evidence highlights the urgent need for transformative approaches to midwifery training, ensuring that midwives and other healthcare professionals (nurses, doctors, and community health workers) integrate elements of midwifery care into their practice. This approach enhances women's capabilities, fosters critical thinking, supports decision-making, prevents unnecessary interventions, and facilitates appropriate responses to emergencies. The studies included in this research topic are consistent with evidence indicating persistent gaps in SRH and rights. Addressing these gaps requires improving midwifery education and research, strengthening postgraduate studies, scaling up midwifery services, and advancing continuous education for midwives.
